# Maximum intensity projection based on high frame rate contrast-enhanced ultrasound for the differentiation of breast tumors

**DOI:** 10.3389/fonc.2023.1274716

**Published:** 2023-10-30

**Authors:** Jia Li, Cong Wei, Xinxin Ma, Tao Ying, Di Sun, Yuanyi Zheng

**Affiliations:** Department of Ultrasound in Medicine, Shanghai Sixth People’s Hospital Affiliated to Shanghai Jiao Tong University School of Medicine, Shanghai, China

**Keywords:** breast tumors, maximum intensity projection, contrast-enhanced ultrasound, angiogenesis, microvasculature

## Abstract

**Objective:**

We explored the role of maximum intensity projection (MIP) based on high frame rate contrast-enhanced ultrasound (H-CEUS) for the differentiation of breast tumors.

**Methods:**

MIP imaging was performed in patients with breast tumors who underwent H-CEUS examinations. The microvasculature morphology of breast tumors was assessed. The receiver operating characteristic curve was plotted to evaluate the diagnostic performance of MIP.

**Results:**

Forty-three breast tumors were finally analyzed, consisting of 19 benign and 24 malignant tumors. For the ≤30-s and >30-s phases, dot-, line-, or branch-like patterns were significantly more common in benign tumors. A tree-like pattern was only present in the benign tumors. A crab claw-like pattern was significantly more common in the malignant tumors. Among the tumors with crab claw-like patterns, three cases of malignant tumors had multiple parallel small spiculated vessels. There were significant differences in the microvasculature morphology for the ≤30-s and >30-s phases between the benign and malignant tumors (all *p* < 0.001). The area under the curve, sensitivity, specificity, accuracy, positive predictive value, and negative predictive value of the ≤30-s phase were all higher than those of the >30-s phase for the classification of breast tumors.

**Conclusion:**

MIP based on H-CEUS can be used for the differentiation of breast tumors, and the ≤30-s phase had a better diagnostic value. Multiple parallel small spiculated vessels were a new finding, which could provide new insight for the subsequent study of breast tumors.

## Introduction

Currently, breast cancer has become the most common malignant tumor instead of lung cancer in 2020 and is the main cause of tumor-related mortality threatening women’s health worldwide (1). Angiogenesis, or new vessel formation, is essential for tumor development and metastasis (2, 3) because it can offer the necessary oxygen and nutrients for tumor progression. Angiogenesis is considered to provide valuable information for the differential diagnosis of breast tumors. Vessel morphology in malignant breast tumors is significantly different compared with benign tumors, with malignant tumors tending to have a chaotic pattern, irregular branch pattern, and penetrating vessel in the peripheral region of the tumor (4). Thus, assessing angiogenesis is important to distinguish benign from malignant breast tumors.

To evaluate angiogenesis in breast tumors, a variety of imaging technologies, such as ultrasound and magnetic resonance imaging (MRI), are used in clinical practice. Ultrasound examination is usually applied as an initial screening imaging modality for breast diseases in China. For conventional ultrasound, color Doppler flow imaging (CDFI) and power Doppler imaging (PDI) are widely available techniques for the evaluation of breast tumor angiogenesis. However, the ability of CDFI or PDI to assess angiogenesis is limited because they are only sensitive to fast flow, making it difficult to image microvasculature (5). Significant overlaps in vessel features are revealed in discriminating malignant from benign breast tumors using CDFI or PDI (6, 7). Compared with CDFI and PDI, contrast-enhanced ultrasound (CEUS) and MRI have the advantage of evaluating tumor angiogenesis. They can both image tumor microvasculature with the help of contrast agents. However, CEUS and MRI have difficulty in clearly depicting microvascular structure due to limitations in resolution (8, 9). Therefore, it is necessary to find a better diagnostic approach to image microvasculature.

Maximum intensity projection (MIP) is an accumulated imaging technology with high resolution (10) that images microvasculature on the basis of CEUS. MIP is capable of reconstructing microvasculature images by tracing microbubbles in consecutive CEUS images, thus showing the microvasculature course (10–13). MIP has superior capability for detecting microbubbles, despite the low number of microbubbles and low-flow microbubbles. To date, this technology has been applied in human beings, such as for the liver, prostate, and breast. It has been reported that MIP can show fine vessel structure in human tumors (10, 13–15). With respect to breast tumors, a previous study revealed that MIP could more clearly depict the microvasculature structure of breast tumors than CEUS and contribute to distinguishing benign from malignant breast tumors (14). However, MIP in that study is based on conventional frame rate CEUS. The frame rate (FR) is defined as the number of frames per second for ultrasound images. Currently, the FR of conventional CEUS for breasts is mainly below 13 frames per second, which is not sufficient to capture fast-moving microbubbles in some breast tumors with hypervascularity. This leads to partial loss of microvasculature information of MIP based on CEUS, thus affecting the vasculature morphology of breast tumors. However, vasculature morphology is considered an important indicator for the differentiation of breast tumors (16, 17). Thus, the FR of CEUS needs to be improved. High frame rate CEUS (H-CEUS) increases temporal resolution by improving FR, which can better capture fast-moving microbubbles and aid the differential diagnosis of breast tumors. At present, few studies have been conducted to investigate MIP based on H-CEUS to differentiate breast tumors.

Therefore, we explored the role of MIP based on H-CEUS for the differentiation of breast tumors.

## Materials and methods

### Patients

**Figure 1:**
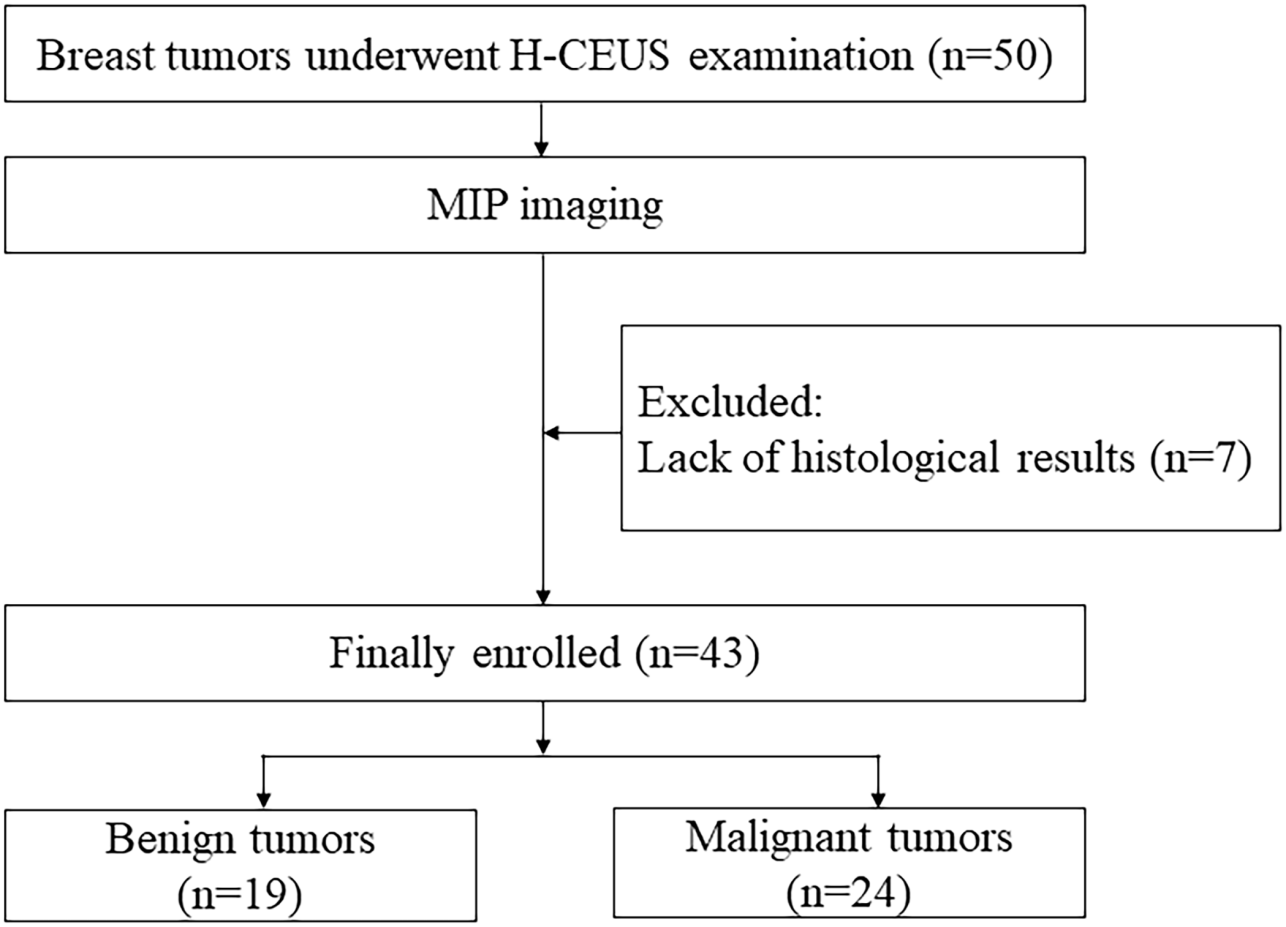
A flowchart presenting the recruitment of patients is shown.

Between August 2021 and January 2023, 41 patients with 50 breast tumors underwent H-CEUS examinations in our hospital. MIP imaging was performed in patients with breast tumors. The inclusion criteria were as follows: a) adults aged ≥18 years, b) all sexes, and c) CEUS examination before biopsy or surgery. The exclusion criteria were the lack of pathologic results. Eventually, 32 patients with 43 breast tumors were enrolled in this study. A flowchart presenting the recruitment of patients is shown in **Figure 1**.

### Conventional ultrasound and CEUS examinations

A Philips EPIQ Elite (Philips Healthcare, Bothell, WA, USA) equipped with an eL18-4 (4–18 MHz) linear transducer or a Resona 9 (Mindray, Shenzhen, China) equipped with L11-3U (11 MHz) or L9-3U (9 MHz) linear transducer was applied for conventional ultrasound and CEUS examinations. All patients were examined by experienced sonographers. First, a grayscale ultrasound was performed to identify the breast tumor. Subsequently, CDFI was performed to assess blood flow within and outside the breast tumor in different sections. Ultimately, the section that revealed the richest blood flow was chosen for the CEUS examination based on CDFI. During the conventional ultrasound and CEUS examinations, the transducer was gently placed on the skin to avoid vessel squeezing, and the patients were instructed to maintain their posture and respire calmly. A low mechanical index of less than 0.1 and a frame rate of 23–32 frames per second affected by image depth were used for the CEUS mode. The parameters were unchanged throughout the examination. CEUS was performed with the acoustic contrast agent SonoVue (Bracco, Milan, Italy). Each patient was injected with a dose of 0.5 ml as a bolus via a forearm vein, followed by flushing with 5 ml saline. At the same time, the timer on the machine was started, and 3 min of CEUS dynamic images was captured after bolus injection.

### MIP imaging

With a CEUS time cutoff criterion of 30 s, the MIP time was classified into two phases: ≤30-s phase (from the injection of the contrast agent to CEUS time of 30 s) and >30-s phase (from CEUS time of 31 s to the disappearance of the contrast agent). The data from the CEUS dynamic images of each phase were respectively post-processed offline by MATLAB R2021a software (The MathWorks, Natick, MA, USA), and multiple MIP images stacked by every 5th frame were obtained. The MIP image in which the microvasculature was richest and clear for each phase was analyzed.

### MIP evaluation

The same two sonographers evaluated the MIP images. If the result was inconsistent, a third sonographer evaluated the MIP images. Three sonographers discussed the final images until a consensus was reached. All three sonographers were unaware of the pathology, clinical histology, and other imaging features.

On MIP images, the microvasculature morphology of breast tumors in each phase was assessed. The microvasculature morphology was classified into three patterns: 1) dot-, line-, or branch-like pattern; 2) tree-like pattern; and 3) crab claw-like pattern. Dot-, line-, or branch-like patterns were defined as dot or linear (straight or curved) vessels, with/without peripheral annular vessels and with/without branching vessels. A tree-like pattern was defined as a main vessel emitting multiple branches similar to a tree. A crab claw-like pattern was defined as radial vessels (more than 2), with/without multiple parallel small spiculated vessels in the peripheral region.

### Statistical analysis

A Shapiro–Wilk test was utilized to examine if continuous variables had a normal distribution. Age with a normal distribution was described using the mean ± standard deviation. Categorical variables were described using frequencies and percentages. Fisher’s exact test was utilized to compare the microvasculature morphology in each phase. With the pathology results as the gold standard, a receiver operating characteristic (ROC) curve was plotted to assess the diagnostic performance of MIP in each phase. The area under the curve (AUC), sensitivity, specificity, accuracy, positive predictive value (PPV), and negative predictive value (NPV) were computed and expressed with a 95% confidence interval (CI). A *p*-value of <0.05 was considered statistically significant. Software SPSS version 25.0 and MedCalc version 20.0.3 were used for all statistical analyses.

## Results

### Clinical features

Forty-three breast tumors confirmed by pathological examination after either needle biopsy or surgical biopsy were finally analyzed, consisting of 19 benign and 24 malignant tumors. All the patients were women, and the median age was 50.41 ± 17.40 years (range, 22–81 years). **Table 1** presents the detailed pathologic results of breast tumors.

**Table 1:** The detailed pathologic results of breast tumors (*n* = 43).

Breast tumors	Number/percentage
Benign	19 (44%)
Fibroadenoma	10
Intraductal papilloma	4
Adenosis	4
Benign phyllodes tumor	1
Malignant	24 (56%)
Invasive breast carcinoma	21
Ductal carcinoma *in situ*	2
Mucinous carcinoma	1

### The patterns of microvasculature morphology on MIP in each phase between benign and malignant breast tumors

**Figure 2:**
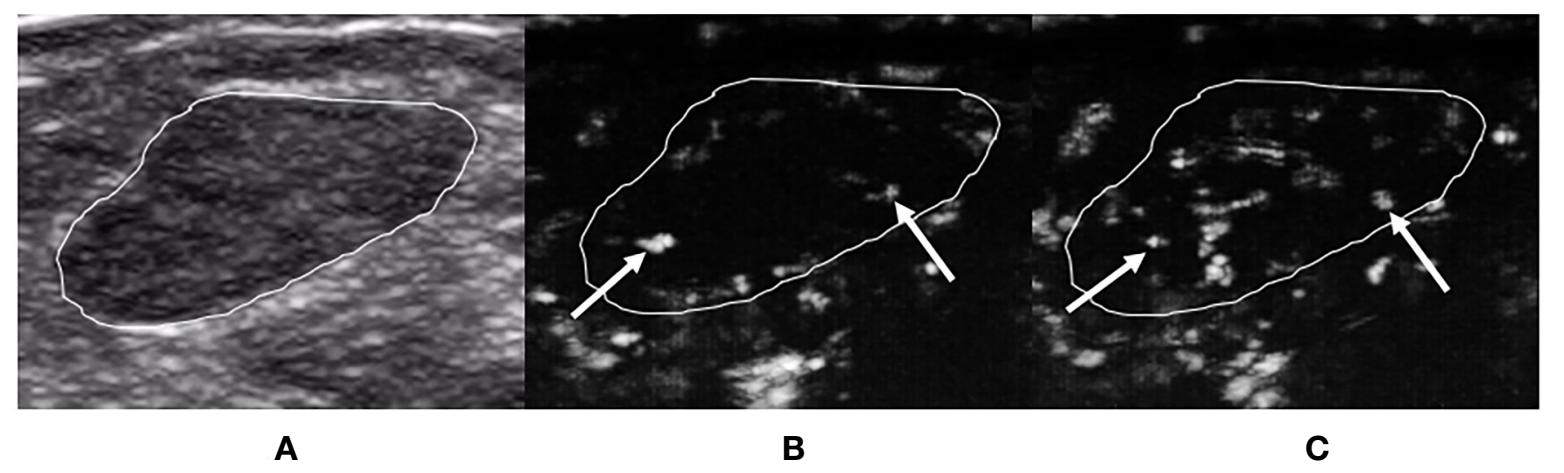
A 29-year-old woman with breast fibroadenoma proven by pathology. The tumor region is marked in white. **(A)** Grayscale ultrasound image of breast tumor. **(B)** The ≤30-s phase: microvasculature morphology shows dot-, line-, or branch-like patterns (arrows). **(C)** The >30-s phase: microvasculature morphology shows dot-, line-, or branch-like patterns (arrows).

**Figure 3:**
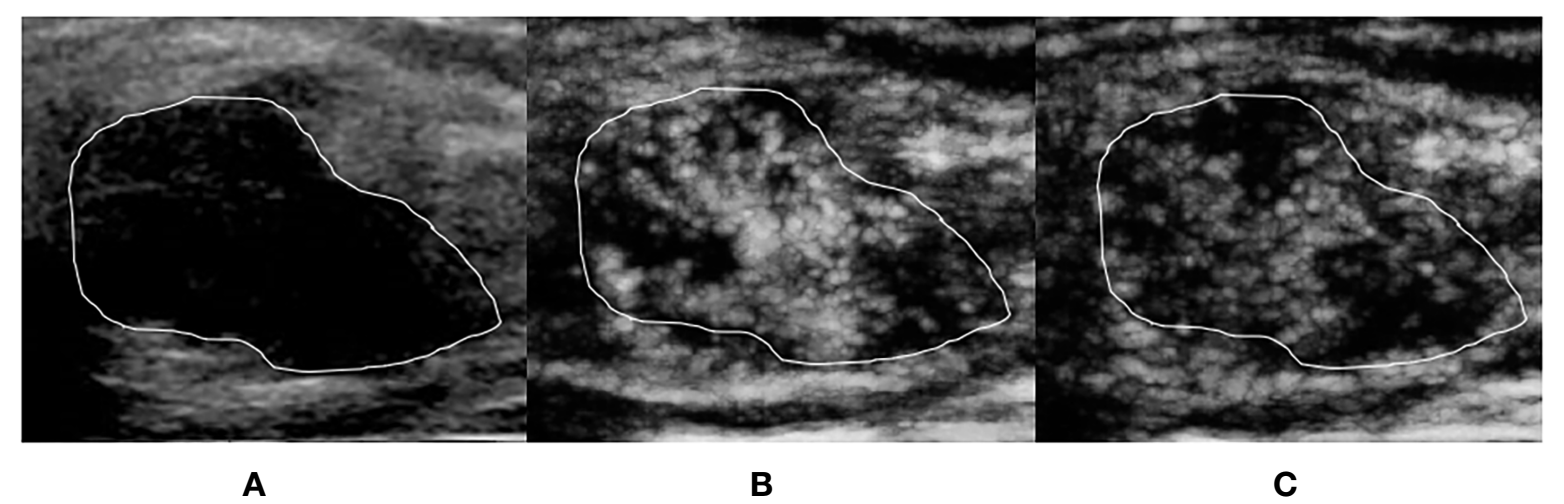
A 39-year-old woman with breast intraductal papilloma proven by pathology. The tumor region is marked in white. **(A)** Grayscale ultrasound image of breast tumor. **(B)** The ≤30-s phase: microvasculature morphology shows a tree-like pattern. **(C)** The >30-s phase: microvasculature morphology shows a tree-like pattern, which is not as clear as the ≤30-s phase.

**Figure 4:**
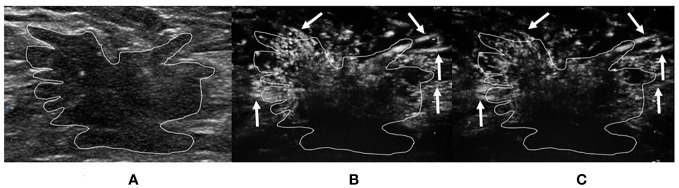
A 40-year-old woman with invasive breast carcinoma proven by pathology. The tumor region is marked in white. **(A)** Grayscale ultrasound image of breast tumor. **(B)** The ≤30-s phase: microvasculature morphology shows a crab claw-like pattern (arrows) in the peripheral region of the tumor. **(C)** The >30-s phase: microvasculature morphology shows a crab claw-like pattern (arrows) in the peripheral region of the tumor.

**Figure 5:**
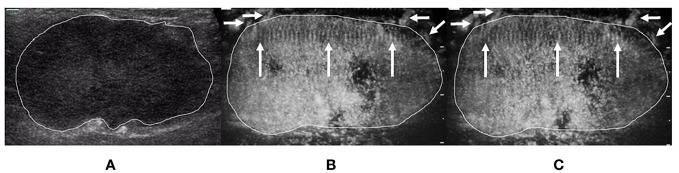
A 75-year-old woman with invasive breast carcinoma proven by pathology. The tumor region is marked in white. **(A)** Grayscale ultrasound image of breast tumor. **(B)** The ≤30-s phase: the tumor increased in scope compared with the grayscale ultrasound. Microvasculature morphology shows a crab claw-like pattern (short arrows), with multiple parallel small spiculated vessels (long arrows) in the peripheral region of the tumor. **(C)** The >30-s phase: the tumor increased in scope compared with the grayscale ultrasound. Microvasculature morphology shows a crab claw-like pattern (short arrows), with multiple parallel small spiculated vessels (long arrows) in the peripheral region of the tumor.

For the ≤30-s phase, dot-, line-, or branch-like patterns (**Figure 2**) were present in 13 benign tumors and 1 malignant tumor. A tree-like pattern (**Figure 3**) was present in five benign tumors, consisting of four cases of intraductal papillomas and one case of adenosis. There was no tree-like pattern in the malignant tumors. A crab claw-like pattern (**Figure 4**) was present in 1 benign tumor and 23 malignant tumors. Among the tumors with crab claw-like patterns, three cases of malignant tumors had multiple parallel small spiculated vessels (**Figure 5**).

For the >30-s phase, dot-, line-, or branch-like patterns were present in 14 benign tumors and 4 malignant tumors. A tree-like pattern was present in three benign tumors, all of which were intraductal papillomas. There was no tree-like pattern in the malignant tumors. The tree-like pattern for the ≤30-s phase was clearer than that of the >30-s phase. A crab claw-like pattern was present in 2 benign tumors and 20 malignant tumors. Among the tumors with crab claw-like patterns, three cases of malignant tumors had multiple parallel small spiculated vessels.

### Comparison of microvasculature morphology on MIP in each phase between benign and malignant breast tumors

For the ≤30-s phase, dot-, line-, or branch-like patterns were significantly more common in benign tumors (68% vs. 4%). A tree-like pattern was only present in benign tumors, as compared with malignant tumors (27% vs. 0%). A crab claw-like pattern was significantly more common in malignant tumors (96% vs. 5%). There was a significant difference in the microvasculature morphology between the benign and malignant tumors (*p* < 0.001) (**Table 2**).

**Table 2:** Comparison of microvasculature morphology in the ≤30-s phase between benign and malignant breast tumors.

Pathology	≤30-s phase [*n* (%)]		
	Dot-, line-, or branch-like pattern	Tree-like pattern	Crab claw-like pattern
Benign (*n* = 19)	13 (68)	5 (27)	1 (5)
Malignant (*n* = 24)	1 (4)	0 (0)	23 (96)
*p*-value	0.000^a*^		

a Fisher’s exact test.

^*^Statistically significant.

For the >30-s phase, dot-, line-, or branch-like patterns were significantly more common in benign tumors (74% vs. 17%). A tree-like pattern was only present in the benign tumors, as compared with the malignant tumors (16% vs. 0%). A crab claw-like pattern was significantly more common in malignant tumors (83% vs. 10%). There was a significant difference in the microvasculature morphology between the benign and malignant tumors (*p* < 0.001) (**Table 3**).

**Table 3:** Comparison of microvasculature morphology in the >30-s phase between benign and malignant breast tumors.

Pathology	>30-s phase [*n* (%)]		
	Dot-, line-, or branch-like pattern	Tree-like pattern	Crab claw-like pattern
Benign (*n* = 19)	14 (74)	3 (16)	2 (10)
Malignant (*n* = 24)	4 (17)	0 (0)	20 (83)
*p*-value	0.000^a*^		

a Fisher’s exact test.

^*^Statistically significant.

### Diagnostic performance of MIP in each phase between benign and malignant breast tumors

**Figure 6:**
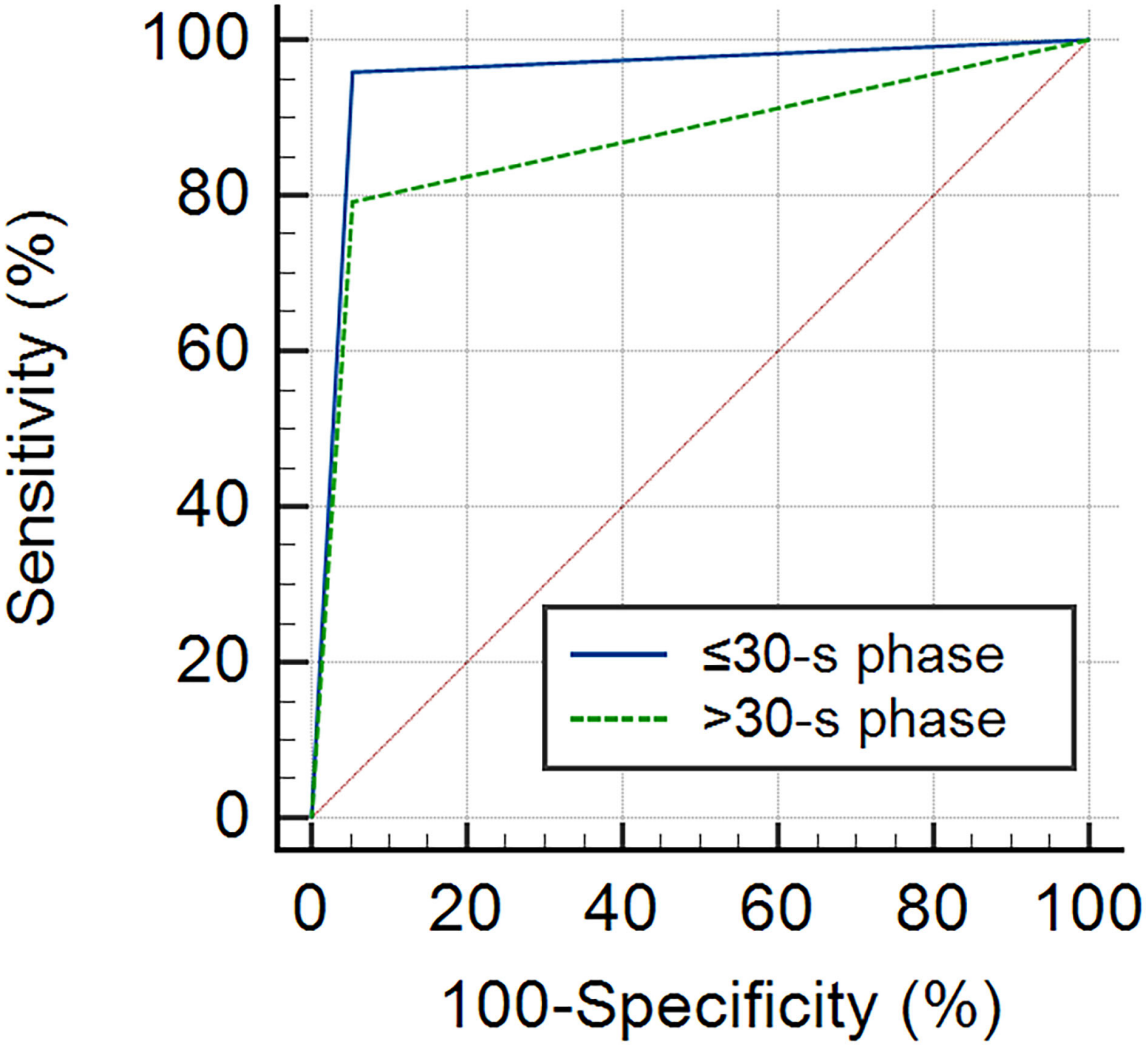
The ROC curve of MIP in each phase for the classification of benign and malignant breast tumors. The AUC of the ≤30-s phase was 0.953 (95% CI: 0.841–0.994), and the AUC of the >30-s phase was 0.864 (95% CI: 0.725–0.949).

According to the ROC curve analysis, the AUC of the ≤30-s phase was higher than that of the >30-s phase for the classification of breast tumors (0.953 vs. 0.864) (**Figure 6**). Benign tumors were based on dot-, line-, or branch-like patterns, while malignant tumors were based on a crab claw-like pattern. The sensitivity, specificity, accuracy, PPV, and NPV of the ≤30-s phase were all higher than those of the >30-s phase for the classification of breast tumors (95.8% vs. 83.3%, 94.7% vs. 89.5%, 95.4% vs. 86.0%, 95.8% vs. 90.9%, and 94.7% vs. 81.0%) (**Table 4**).

**Table 4:** Diagnostic performance of MIP in each phase between benign and malignant breast tumors.

Phase	Sensitivity (%) (95% CI)	Specificity (%) (95% CI)	Accuracy (%) (95% CI)	PPV (%) (95% CI)	NPV (%) (95% CI)
≤30 s	95.8 (78.9–99.9)	94.7 (74.0–99.9)	95.4 (84.2–99.4)	95.8 (77.3–99.4)	94.7 (72.5–99.2)
>30 s	83.3 (62.6–95.3)	89.5 (66.9–98.7)	86.0 (72.1–94.7)	90.9 (72.7–97.4)	81.0 (63.2–91.3)

PPV, positive predictive value; NPV, negative predictive value; CI, confidence interval.

## Discussion

In our study, the value of MIP based on H-CEUS in differentiating benign from malignant breast tumors was assessed. Our results demonstrated that dot-, line-, or branch-like and tree-like patterns were associated with benign breast tumors, whereas the crab claw-like pattern was associated with malignant breast tumors for both the ≤30-s and >30-s phases. We also found that the AUC, sensitivity, specificity, accuracy, PPV, and NPV of the ≤30-s phase were all higher than those of the >30-s phase.

Assessing tumor vessels has been suggested to be helpful for diagnosing, choosing management plans, and predicting the prognosis of malignant breast tumors (6). MIP is a microvascular imaging technology that has been applied to differentiate benign from malignant breast tumors. Du et al. (14) reported that a tree-like pattern was related to benign breast tumors, a crab claw-like pattern was related to malignant breast tumors, and a root hair-like pattern was observed in both benign and malignant breast tumors. Similar to that study, our study revealed that a crab claw-like pattern was more common in malignant breast tumors. Slightly different from that study, our study demonstrated that a tree-like pattern was only present in benign breast tumors. Unlike that study, our study also found that dot-, line-, or branch-like patterns were more common in benign breast tumors, which was not reported in that study. Furthermore, a root hair-like pattern was not observed in our study. The possible explanations for the difficulty were the different observed populations and FRs of CEUS.

It is worth mentioning that multiple parallel small spiculated vessels were found in the peripheral region of a small portion of malignant breast tumors in the present study. To date, no previous studies have reported on this phenomenon. The common feature of these malignant breast tumors was that the histopathological types were all invasive breast carcinomas in the present study. Multiple parallel small spiculated vessels may be associated with angiogenesis at the periphery of the breast tumor. Planeix et al. (18) found that endothelial follicle-stimulating hormone receptor expression in malignant breast tumors was related to angiogenesis in the peripheral region of tumors, which can remodel vessels and form abnormal arterioles and venules. It has been reported that mast cells located in the stroma surrounding a tumor are associated with angiogenesis in malignant breast tumors, which could be involved in inflammatory reactions at the tumor periphery (19, 20). In the future, further investigations to determine the exact mechanisms of multiple parallel small spiculated vessels are needed. Multiple parallel small spiculated vessels had a characteristic microvasculature morphology and were easily recognized, which could provide valuable information for the classification of breast tumors. In addition, this new finding could provide new insight for the subsequent study of breast tumors—for example, whether this microvasculature morphology is related to the clinical stages, certain pathological components, and molecular subtypes of breast cancers.

In this study, with the CEUS time cutoff criterion of 30 s, the MIP time was classified into two phases: ≤30-s phase and >30-s phase. We found that the same breast tumor could show different microvasculature morphologies in different phases of MIP. For example, dot-, line-, or branch-like patterns in benign breast tumors in the >30-s phase were more common than those in the ≤30-s phase (74% vs. 68%); similarly, dot-, line-, or branch-like patterns in malignant breast tumors in the >30-s phase were also more common than those in the ≤30-s phase (17% vs. 4%). In other words, different phases of MIP affected the microvasculature morphology of breast tumors, which could further influence the differential diagnosis of breast tumors. In fact, the amount of contrast agent varies over CEUS time, from none to peak to decline. The reason for this variation in microvasculature morphology may be related to the difference in the amount of contrast agent in different phases of MIP. The AUC, sensitivity, specificity, accuracy, PPV, and NPV of the ≤30-s phase were all higher than those of the >30-s phase for the classification of breast tumors (0.953 vs. 0.864, 95.8% vs. 83.3%, 94.7% vs. 89.5%, 95.4% vs. 86.0%, 95.8% vs. 90.9%, and 94.7% vs. 81.0%). Thus, the ≤30-s phase had a significant advantage for the classification of breast tumors.

Based on the above findings, the results of the ≤30-s phase for the classification of breast tumors were used as the diagnostic performance of our study. In a previous study based on conventional FR CEUS, the sensitivity, specificity, and accuracy were 93.8%, 86.2%, and 90.2%, respectively (14). These findings were all lower than those of our study, indicating that the diagnostic value of MIP based on H-CEUS may be better than that of MIP based on conventional FR CEUS. However, further research is required. The ≤30-s phase had relatively high specificity, which would provide an alternative method for downgrading Breast Imaging Reporting and Data System (BI-RADS) category 4A lesions. In clinical practice, breast lesions are classified into seven categories (categories 0–6) using the BI-RADS. Among them, BI-RADS category 4 is further categorized into three subclasses: 4A, 4B, and 4C. The system has been widely applied in the risk assessment of patients with breast lesions. It is recommended to perform a biopsy for BI-RADS category 4 and 5 lesions (21). However, it is difficult to accurately differentiate benign from malignant lesions for BI-RADS category 4 lesions, which leads to excessive biopsies and surgeries of benign lesions (22). Previous studies have reported that BI-RADS category 4A lesions make up about 50% of BI-RADS category 4 lesions, whereas a small part (7.6%) of the lesions have been shown to be malignant (23, 24). Thus, the proper downgrading of BI-RADS category 4A lesions to BI-RADS category 3 lesions was needed.

Intraductal papilloma is a common benign papillary tumor, representing 5% of benign breast tumors (25). It can occur at any age but is frequently observed in women between 30 and 77 years of age (26). As a benign papillary tumor, intraductal papilloma needs to be differentiated from malignant papillary tumors, such as ductal carcinoma *in situ*, invasive ductal carcinoma, and papillary carcinoma. Furthermore, intraductal papilloma has a higher risk of carcinogenesis (27). Therefore, a correct diagnosis of intraductal papilloma is essential for subsequent treatment. MRI and ultrasound discriminate benign, including intraductal papilloma, from malignant papillary tumors, which is challenging due to overlapping imaging features (28–30). In the current study, we used MIP based on H-CEUS to image the microvasculature of breast tumors. We found that intraductal papillomas all showed tree-like patterns in the ≤30-s phase, accounting for four-fifths of all tree-like patterns. Moreover, the microvasculature morphology of the tree-like pattern was clearer in the ≤30-s phase than in the >30-s phase. This suggests that intraductal papilloma is associated with a tree-like pattern and that the ≤30-s phase could help diagnose this disease. To the best of our knowledge, the relationship between intraductal papilloma and tree-like pattern has not been reported previously on MIP. In the current study, we also found that the microvasculature morphology of malignant breast tumors, including ductal carcinoma *in situ*, had no tree-like pattern. Based on the above results, MIP based on H-CEUS has the potential to differentiate benign and malignant papillary tumors.

There were a few limitations in the current study. First, few studies have previously explored the microvasculature morphology on MIP based on H-CEUS, but the sample size of our research was small, particularly for assessing the diagnostic performance of breast tumors. Second, only two-dimensional imaging was performed in this study. Compared with two-dimensional imaging, three-dimensional imaging can acquire more comprehensive information on vessels in breast tumors. To compensate for this shortage as much as possible on two-dimensional imaging, the section that revealed the richest vessels was chosen for the CEUS examination. Then, MIP based on the CEUS examination was conducted. Third, two ultrasound imaging systems with different central frequencies were employed in this study, and we did not compare the effect of different central frequencies on the MIP image patterns. Theoretically, we think the central frequencies will affect the image quality instead of the patterns of tumor vessels. It is still important that this needs to be investigated in a future study.

In conclusion, MIP based on H-CEUS can be used for the differentiation of benign and malignant breast tumors, and the ≤30-s phase had a better diagnostic value. Multiple parallel small spiculated vessels were a new finding, which could provide new insight for the subsequent study of breast tumors.

## Data availability statement

The raw data supporting the conclusions of this article will be made available by the authors, without undue reservation.

## Ethics statement

The studies involving humans were approved by the Ethics Committee of Shanghai Sixth People’s Hospital Affiliated to Shanghai Jiao Tong University School of Medicine. The studies were conducted in accordance with the local legislation and institutional requirements. The participants provided their written informed consent to participate in this study.

## Author contributions

JL: Conceptualization, Data curation, Formal Analysis, Investigation, Methodology, Visualization, Writing – original draft. CW: Conceptualization, Data curation, Formal Analysis, Methodology, Visualization, Writing – original draft. XM: Data curation, Formal Analysis, Writing – original draft. TY: Conceptualization, Formal Analysis, Methodology, Supervision, Validation, Writing – review & editing. DS: Conceptualization, Methodology, Supervision, Validation, Writing – review & editing. YZ: Conceptualization, Formal Analysis, Funding acquisition, Investigation, Methodology, Project administration, Supervision, Validation, Writing – review & editing.
